# Osteogenic potential of *punica granatum* through matrix mineralization, cell cycle progression and runx2 gene expression in primary rat osteoblasts

**DOI:** 10.1186/s40199-014-0072-7

**Published:** 2014-11-20

**Authors:** Sahabjada Siddiqui, Mohammad Arshad

**Affiliations:** Molecular Endocrinology Laboratory, Department of Zoology, University of Lucknow, Lucknow, 226007 India

**Keywords:** Cell cycle, Osteoblast, Osteogenesis, *Punica granatum*, Runx2

## Abstract

**Background:**

Osteoporosis is one of the prevalent diseases in ageing populations. Due to side effects of many chemotherapeutic agents, there is always a need to search for herbal products to treat the disorder. *Punica granatum* (PG) represent a potent fruit-bearing medicinal herb which exerted valuable anti-osteoporotic activities. The present study was carried out to validate the *in vitro* osteogenic effects of the PG seed extract in primary calvarial osteoblast cultures harvested from neonatal rats.

**Methods:**

The ethanolic extract of PG was subjected to evaluate cell proliferation, regeneration, mineralization and formation of collagen matrix using MTT, alkaline phosphatase, Alizarin Red-S staining and Sirius Red dye, respectively. Cell cycle progression and osteogenic gene Runx2 expression were carried out by flow cytometry and real time PCR, respectively.

**Results:**

Exposure of different concentrations (10–100 μg/ml) of the extract on osteoblastic cells showed characteristic morphological changes and increment in cell number. A significant growth in cell proliferation, ALP activity, collagen contents and matrix mineralization of osteoblasts in a dose dependent manner (p < 0.05), suggested that PG has a stimulatory effect on osteoblastic bone formation or potential activity against osteoporosis. In addition, PG extract also enhanced DNA content in S phase of cell cycle and Runx2 gene expression level in osteoblasts.

**Conclusion:**

The data clearly indicated that PG promoting bone cell proliferation and differentiation in primary osteoblasts might be due to elevating the osteogenic gene Runx2 expression. The present study provides an evidence for PG could be a promising herbal medicinal candidate that able to develop drugs for osteoporosis.

## Introduction

Osteoporosis is a metabolic bone disorders that afflicts about 200 million people worldwide. It is mainly prevalent in women (approximately 80%) and also older men [1]. The bone remodeling process is the alternative of this severe concern, and it’s dependable for repair of damage or formation and resorption of bones to maintain the integrity of the skeleton. However, any abnormalities in remodeling that lead to fracture in the bones and osteoporosis.

Recently, several conventional and antiresorptive drugs are used to reduce fracture risk in osteoporosis including hormone replacement therapy (HRT), bisphosphonates, selective estrogen receptor modulators (SERMs) and calcitonin [2]. However, these drugs and therapy have multiple side effects, which causes heart related issues, headache, dizziness, anorexia, cramping of legs and gastrointestinal related problem, particularly pain in stomach and heartburn [3]. The research still continues for the enhancement of such benefit to lower risk involved in human beings.

Anabolic or osteogenic therapies are preferred for pharmacological development to treat osteoporosis [4]. Parathyroid hormone (PTH 1–34) only anabolic agent for the treatment of postmenopausal osteoporosis that is also recommended by the FDA (Food and Drug Administration) which regulates the formation of bones by enhancing the cell proliferation of the osteoblastic lineage or inducing differentiation of osteoblast progenitor cells. Whilst, this therapy is also related to an increased risk of cancer, such as osteosarcoma [5,6].

In the recent time, several medicinal plants are used for health care treatments and management especially bone related diseases. Although compared with other drug treatments, herbal products create no/little side effects. *Punica granatum* (Pomegranate, PG) is one of the most potent fruit-bearing medicinal herbs widely distributed throughout the Mediterranean region of southern Europe, northern Africa and tropical Africa, Indian subcontinent, Central Asia and the drier parts of South-East Asia. PG seeds contain punicic acid, ellagic acid, steroidal estrogen and non-steroidal phytoestrogens, including comesten, coumoestrol and isoflavones genistein, daidzein and ascorbic acids. PG also contains estrogenic compound such as luteolin, quercetin, kaempferol, estrone and estradiol, which are responsible for bone formation and also inhibit the resorption process [7-12]. A non-isoflavone phytoestrogenic compounds such as quercetin, rutin, apigenin and coumestrol has also been reported in various legumes [13]. A crude PG extract and its seed oil enhance bone healing properties and prevent loss of bones because of the proliferation of osteoblast, inhibition of osteoclast cell and also decrease the inflammation. [14,15]. Recently, PG has been also utilized to inhibit acetyl cholinesterase activity as a new source for management of Alzheimer’s disease [16].

Osteoblast differentiation and proliferation mediated by different growth factors such as bone morphogenetic proteins (BMPs), transforming growth factor beta (TGFβ) and core-binding factor alpha1 (Cbfα1) are known to be targeted the osteogenic Runt-related transcription factor2 (Runx2) gene [17]. Runx2 is the key transcription factor initiating and regulating the early osteogenesis and late mineralization of bone. Furthermore, Runx2 triggers the expression of major bone matrix genes during the early stages of osteoblast differentiation [18].

There is no evidence regarding the investigation of the PG seed extract on bone cell proliferation, differentiation, and collagen matrix formation in primary culture of osteoblasts. The present study also described the matrix mineralization activity along with osteogenic gene Runx2 expression by real time PCR and DNA content analysis in the S phase of the cell cycle in osteoblastic cells. The findings suggested that PG may be a potent osteogenic herbal drug that induces bone cells proliferation and regeneration following increased DNA contents and Runx2 gene expression which provide future prospects in the development of anti-osteoporotic drugs and therapy.

## Materials and methods

### Reagents and chemicals

Alpha modified minimum essential medium (α-MEM), fetal bovine serum (FBS), MTT (3-(4,5-dimethylthiazol-2-yl) -2,5-diphenyltetrazolium bromide) dye, *p*-nitrophenyl phosphate (pNPP), naphthol AS­MX phosphate, fast blue BB salt, ascorbic acid and Sirius Red dye were purchased from Himedia, India. β-glycerophosphate, Ribonuclease (RNase) A and propidium iodide (PI) were purchased from Sigma-Aldrich, USA. RNAiso Plus reagent was procured from Takara, India. cDNA synthesis kit was purchased from Thermo Scientific, USA and SYBER green kit from Roche, USA. All the reagents used were of high purity grade.

### Plant materials and extraction

The fresh cultivated PG plant was collected from nearby University of Lucknow, Lucknow, India. Plant materials were identified and authenticated from Department of Pharmacognosy, Faculty of Pharmacy, Integral University, Lucknow. A reference specimen (voucher No. IU/PHAR/HRB/14/08) has been deposited in the herbarium of Faculty of Pharmacy, Integral University, Lucknow. Seed parts of collecting plant were air dried in the shade and crushed to a powder in a mechanical grinder. The 95% ethanolic extract of PG was prepared with the help of Soxhlet apparatus (Borosil Glass Works Limited, India) at 60°C and Whatman No. 1 filter paper was used to obtain filtrate of extract. The filtrate was concentrated in vacuum at 40°C using a rotary evaporator (BUCHI Rotavapor R-205, Switzerland).

### Primary culture of osteoblasts

Osteoblastic cells were isolated from neonatal rat pups calvaria using sequential digestion with slight modification [19]. Briefly, calvaria were dissected from four to five neonatal (1–2 days old) rat pups. After removal of sutures and adherent mesenchymal tissues, calvaria were subjected to five sequential (10–15 min) digestions at 37°C in shaking water bath at 120 rpm containing each of 0.1% dispase and collagenase type II enzymes. Supernatants were pooled from the second to fifth digestions in a tube containing 800 μl FBS. Cells were re-suspended in α-MEM containing 10% FBS with 1% penicillin/streptomycin solution and transfer in T-25 cm^2^ culture flasks. The flasks were incubated at 37°C with 5% CO_2_ in CO_2_ incubator (Excella ECO-170, New Brunswick). The study was approved by the Institutional Animal Ethics Committee of Azad Institute of Pharmacy and Research, Lucknow (Ref. No.: AIPR/2013-14/1398).

### Cell proliferation

The proliferative effect of PG extract was determined using MTT assay with some modification [20]. Calvarial osteoblasts were suspended in α-MEM medium and plated at a density of 2 × 10^3^ cells/well in a 96 wells plate. After overnight incubation, the medium was replaced with a medium containing PG extract solution prepared in media with different concentrations (0, 10, 25, 50 and 100 μg/ml) in triplicate for 48 h. 17β-estradiol (E2) at the concentration of 1 nM was used as a positive control. After exposure period, 10 μl of 5 mg/ml MTT solution was added to each wells and further incubated at 37°C. After 4 h, medium was discarded and 100 μl of dimethyl sulphoxide (DMSO) was added to solubilise the dark blue formazan crystals at 37°C for 10 min. Absorbance was recorded at 540 nm with microplate reader (BIORAD-680, USA) and the percentage viable cells were calculated using the formula:

$$ \%\;\mathrm{Cell}\ \mathrm{viability}=\left[\left(\mathrm{O}\mathrm{D}\ \mathrm{of}\ \mathrm{treated}\right)\;/\;\left(\mathrm{O}\mathrm{D}\ \mathrm{of}\ \mathrm{control}\right)\right]\times 100 $$

The cellular morphology was also observed in other sets of PG treatment under trinocular inverted phase contrast microscopy (Nikon ECLIPSE T*i*-S, Japan).

### Alkaline phosphatase (ALP) activity

ALP assay is based on the hydrolysis of pNPP by ALP into a yellow colored product at alkaline pH. ALP activity of osteoblasts was determined with a slight modification [21]. A 100 μl of cell suspension containing 2 × 10^3^cells /well was seeded in 96-well plates using α-MEM supplemented with 10% FBS, 10 mM β-glycerophosphate, 50 μg/ml ascorbic acid and 1% penicillin/streptomycin (osteoblast differentiation medium) and treated with different concentrations (10–100 μg/ml) of the extract for 48 h. E2 at the concentration of 1 nM was used as a positive control. After completion of incubation period, osteoblast cultures were fixed in 4% paraformaldehyde and stained with a solution containing 0.1 mg/ml naphthol AS­MX phosphate, 0.5% N, N- dimethylformamide, 2 mM MgCl2, and 0.6 mg/ml of fast blue BB salt in 0.1 mM Tris–HCl (pH 8.5) for 20 min. The formation of color was examined and images were taken under an inverted phase contrast microscope. For the quantitative estimation of ALP, the plate was fixed and kept in −70°C for 20 min, and then brought to 37°C for freeze fracture. Next, 50 μl of chilled *p*-nitrophenyl phosphate (pNPP) substrate was added to each wells and incubated at 37°C for 30 min for color development. The absorbance was measured at 405 nM using an ELISA reader.

### Assessment of collagen deposition

Sirius Red is an anionic dye that binds strongly to collagen molecules. Collagen deposition was quantified using Sirius Red dye following slightly modification [22]. Treated cells were washed with PBS and dried at 37°C in 96-wells plate for overnight incubation and then stained with 20 μl of Sirius Red dye (0.1% in saturated picric acid) for 1 h with mild shaking. Sirius Red dye solution (pH 3.5) was prepared in saturated aqueous picric acid (1.3% in H_2_O) at a concentration of 0.1 mg/ml. The stained cell layers were extensively washed with 0.01 N HCl to remove all non-bounded dye. After rinsing, photographs were taken under inverted phase contrast microscope. For quantitative analysis, the stained cells were dissolved in 0.2 ml 0.1 N NaOH at shaker for 30 min. Next, absorbance was measured colorimetrically at 550 nm against 0.1 N NaOH serve as a blank.

### Mineralization assay

Alizarin Red S, an anthraquinone derivative, was used to identify calcium content in osteoblasts according to a method reported previously [23]. Approximately, 2 × 10^4^ cells/well were seeded in 12-wells culture plate in osteoblast differentiation medium with 10^−7^ M dexamethasone. Cells were treated with PG extract at various concentrations (10–100 μg/ml) for 21 days and the medium was changed every alternate day. At the end of the experiment, cells were washed with PBS and fixed with 4% paraformaldehyde in PBS for 15 min. The fixed cells were stained with 40 mM Alizarin Red-S (pH 4.5) for 30 min followed by washing with distilled water. Calcified nodules appearing as bright red color were photographed under inverted phase contrast microscopy. For quantification of staining, 100 mM cetylpyridinium chloride solution was added for 1 h in each well to solubilise and to release calcium-bound alizarin red into solution. A 100 μl of the supernatant from each well were transferred in 96 well plate in triplicate and the absorbance was recorded at 570 nm by a microplate reader.

### Cellular DNA content

Cell cycle phase distribution with cellular DNA content was analyzed using flow cytometry with some modification [24]. Osteoblasts were planted in 6-wells plate at a density 1 × 10^6^ cells/well and treated with different concentrations (10–100 μg/ml) of extract for 48 h. E2 at the concentration of 10 nM was used as a positive control. Cultured cells were washed with cold PBS and fixed in 70% ethanol at −20°C for 2 h. Fixed cells were treated with RNase A (10 mg/ml) and stained with PI dye in the dark for 30 min at room temperature. The PI dye fluorescence of individual nuclei was measured by using a flow cytometer (BD FACS Calibur, Becton Dickinson, USA) and data were analyzed using Cell Quest Pro V 3.2.1 software (Becton Dickinson, USA).

### Quantitative real-time PCR (qPCR)

The total RNA was isolated from cultured osteoblasts treated with PG extract using RNAiso Plus reagent. Aliquots of 2.0 μg of total RNA in a 10 μl reaction volume were subjected to PCR using a cDNA synthesis kit. Quantitative real-time PCR was performed in light cycler PCR system (LightCycler 480, Roche, USA) using SYBER green kit following manufacturer’s instruction. Runx2 gene expression in calvarial osteoblasts was determined by qPCR using an optimized protocol [25]. Sequence of primer pairs of the genes used in the present study were; runx2- CCACAGAGCTATTAAAGTGACAGTG (F), AACAAACTAGGTTTAGAGTCATCAAGC (R) and GAPDH (housekeeping gene) - CAGCAAGGATACTGAGAGCAAGAG (F), GGATGGAATTGTGAGGGAGATG (R). All the data were normalized to GAPDH expression to study the relative expression of the targeted gene.

### Statistics

All results were represented as the means ± SEM of results from all replicates and statistically significance was determined by one-way analysis of variance (ANOVA) followed by Dunnett’s multiple comparison tests. Probability values of p < 0.05 were considered to be statistically significant. All analysis was conducted using the Graph Pad Prism (Ver. 5.1) software.

## Results and discussion

### Microscopic observation of osteoblastic cells

**Figure 1:**
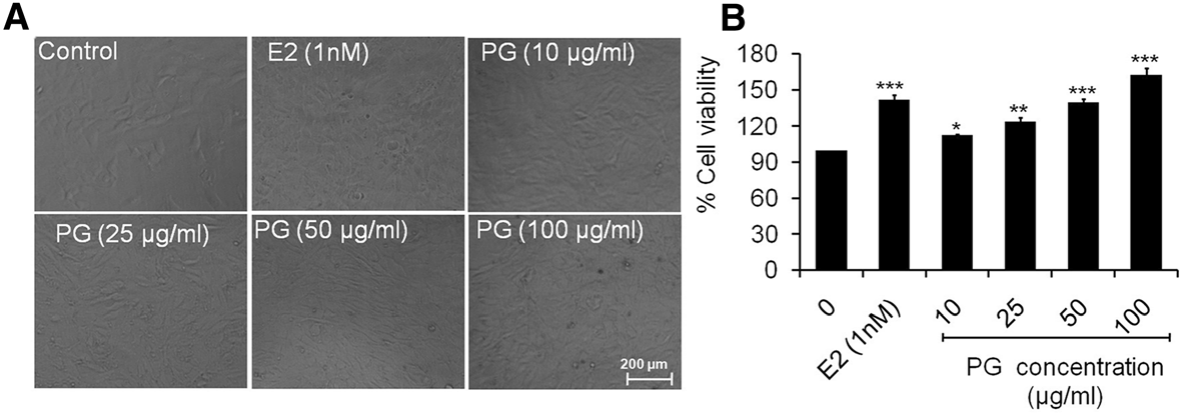
**Percent cell proliferation of primary osteoblasts. (A)** Morphology of osteoblasts under inverted phase contrast microscope treated with different concentrations of PG extract at 48 h **(B)** Percent cell proliferation of osteoblasts treated with 10–100 μg/ml of PG extract. Values were obtained from three independent experiments and expressed as mean ± SEM. ^*^p < 0.05, ^**^p < 0.01 and ^***^p < 0.001 as compared with control.

The morphological changes of untreated (control) and treated osteoblasts with different concentrations of PG extract at 48 h are shown in Figure 1A. The typical spindle shaped with fibroblastic appearance was observed in control under inverted phase contrast microscope. The concentrations 10 and 25 μg/ml of extract enhanced the cell proliferation, moreover, 50 and 100 μg/ml of PG showed the more dense appearance with increased number of cells as compared to control that attributed differentiation of osteoblastic cells. The results revealed that cells exposed to PG extract induce differentiation of osteoblasts as a function of dose due to their morphological alterations and number of cells increment [26].

### Effect of PG on the cell proliferation of osteoblasts

The effect of different doses (10–100 μg/ml) of PG extract on osteoblastic cell proliferation was tested at 48 h (Figure 1B). As compared to control group (cells without extract treatment), PG significantly increased the cell proliferation to 13.03 (p < 0.05) and 24.28% (p < 0.001) at 10 and 25 μg/ml, respectively. Moreover, 50 and 100 μg/ml of PG extract induced cell proliferation to 39.64 and 62.59% (p < 0.001) respectively. The results revealed that PG extract induced the significant cell proliferation in a dose dependent manner. Exposure of cells to 1 nM of 17β-estradiol as a positive control, increased the cell proliferation to 41.72% (p < 0.001) as compared to control. The proliferative effects of PG extract might be due to their estrogenic nature of its contents, including quercetin, kaempferol, estrone and estradiol, which promote bone cell proliferation [11]. A study has shown that PG promoted osteoblast MC3T3-E1 cell proliferation up to approximately 2-fold at 250 μg/ml of plant extracts [7]. Both osteoblast and MCF-7 (human breast adenocarcinoma) cells are an estrogen receptor positive (ER^+^) cells. A similar study has also revealed that MCF-7 cells exposed to legume extracts containing quercetin, daidzein, genistein and kaempferol glycosides at various concentrations (1–1000 μg/ml) showed a maximum cell proliferation at 100 μg/ml of the extracts [27].

### Effect of PG on ALP activity of osteoblasts

**Figure 2:**
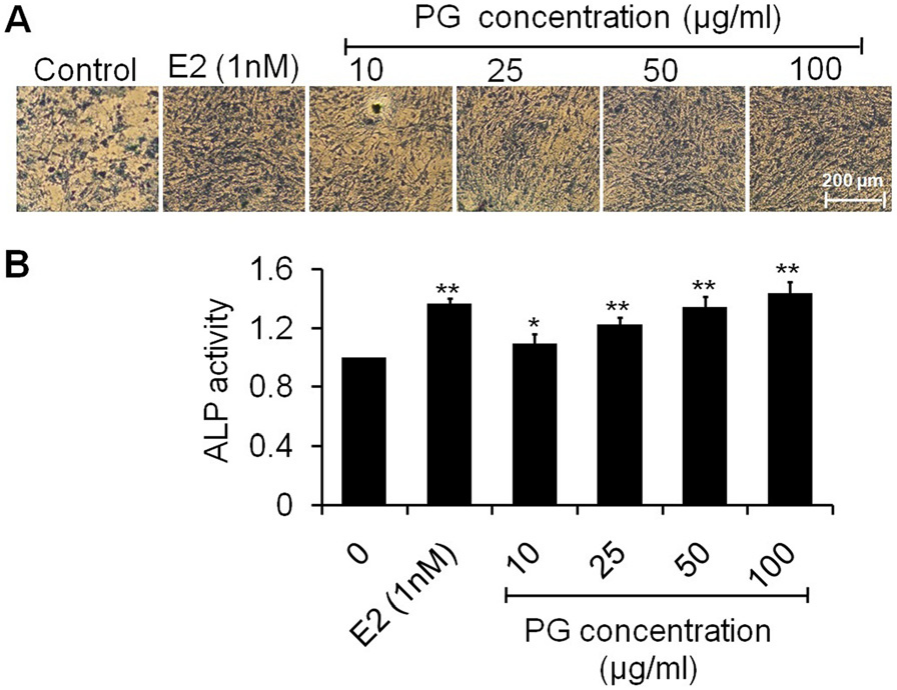
**Measurement of ALP activity. (A)** Photomicrographs stained with fast blue BB salt-ASMX-phosphate complex showing increased formation of ALP in osteoblasts treated with increasing concentrations (10–100 μg/ml) of PG extract at 48 h **(B)** Quantitative data of ALP level presented in the form of ALP activity relative to control. Values are obtained from three independent experiments and expressed as mean ± SEM. ^*^p < 0.05 and ^**^p < 0.01 as compared with control.

Quantitative estimation of alkaline phosphatase activity is one of the biochemical methods, which described the early cell differentiation of osteoblastic cells [28]. Fast blue BB salt-ASMX-phosphate complex acted on ALP activity which appeared to be blue in color. The qualitative data showed that PG extract stimulated ALP stain by increasing the rate of osteoblast cell differentiation in a dose dependent manner (Figure 2A). As observed from numerical data (Figure 2B), concentrations 10 and 25 μg/ml of extract induced ALP level to 9.66% (p < 0.05) and 22.49% (p < 0.01) significantly as compared to control. Also, 50 and 100 μg/ml of extract induced the significant ALP level to 34.67 and 43.95% (p < 0.01) respectively as compared to control. Exposure of cells to 1 nM of E2 increased ALP activity to 36.66% (p < 0.01) as compared to control. Results of ALP assay were the consistent with MTT assay data which suggested that cell proliferation also correlate with cell differentiation. These results indicate that PG extract induces regeneration of osteoblasts as a function of dose might be due to the presence of estrogenic compounds. PG containing estrogenic compounds daidzein and genistein has been reported to possess stimulatory effects synthesizing alkaline phosphatase by osteoblasts *in vitro* [29].

### Effect of PG on collagen deposition by osteoblasts

**Figure 3:**
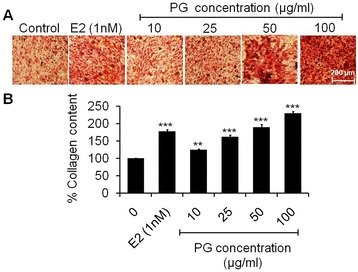
**Collagen formation assay of osteoblasts. (A)** Photomicrographs stained with Sirius Red dye showing increased formation of collagen matrix of osteoblasts treated with increasing concentrations (10–100 μg/ml) of PG extract at 48 h **(B)** Quantitative data of collagen content expressed in the form of percent collagen content respective to control. Data represented as mean ± SEM of three independent experiments. ^**^p < 0.01 and ^***^p < 0.001 as compared with control.

Sirius Red stain was used to assess the extent of collagen (predominantly collagen type I and III fibers) deposited by osteoblasts which developed dense and cross-linked collagen. Collagen comprises 85-90% of the total organic bone matrix [30]. Histological analysis showed that PG extract increases collagen content in osteoblasts in a dose dependent manner as compared to control. PG extract exposed on cells was able to enhance in the collagen density significantly as evident by dark red clusters of collagen evenly distributed throughout the stimulated region (Figure 3A). Further, quantitative measurement of the Sirius Red staining intensity in osteoblasts culture showed a significant increment of 24.96 (p < 0.01), 62.40, 89.37 and 129.84% (p < 0.001) of collagen secretion at 10, 25, 50 and 100 μg/ml of PG exposure, respectively as compared to control (Figure 3B). Exposure of osteoblasts to 1 nM of E2 increased the collagen content to only 77.95% (p < 0.001) as compared to control. Bone shows a variety of structural organizations which is related to the balance between the amount of collagen and mineral [31]. However, not only mineral matrix deposition is essential to ensure a healthy bone, giving strength and rigidity in the skeletal system, but also the adequate deposition of organic matrix (collagen) contributes to bone architecture. Therefore, experiments were conducted to determine whether PG extract exposure enhances the organic matrix deposition. PG treated osteoblasts progressively deposited more collagen (Figure 3A and B) suggesting the collagen deposition induced by PG is a function of dose. A study has reported that ascorbic acid stimulates the formation of collagen matrix at multiple levels, including gene expression, hydroxylation of proline and lysine in collagen during post-translational modification [32].

### Effect of PG on osteoblast mineralization

**Figure 4:**
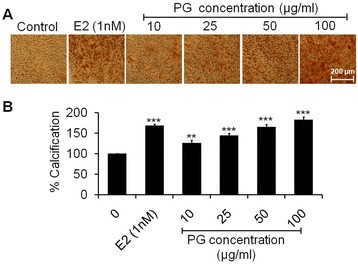
**Mineralization assay of osteoblasts. (A)** Photomicrographs stained with Alizarin Red S showing increased formation of mineralized nodules of osteoblasts treated with increasing concentrations (10–100 μg/ml) of PG extract at 21 days **(B)** Quantitative data of Alizarin Red-S extraction expressed in the form of percent calcification. Data represented as mean ± SEM of three independent experiments. ^**^p < 0.01 and ^***^p < 0.001 as compared with control.

Mineralized nodule formation describes the final stages of osteoblastic differentiation at 21 days. As observed from photomicrographs, osteoblastic cells in proliferation period exhibited a fibroblastic morphology monolayer in control cells. As the progress in mineralization, the cells continued growing slowly and formed a mosaic like multiple layers which was greater at 100 μg/ml of PG extract (Figure 4A). The percent calcification data of Alizarin stain showed that treatment of osteoblasts at 10, 25, 50 and 100 μg/ml of extract significantly increased mineralized nodule formation that signifying by 26.15 (p < 0.01), 44.55, 65.64 and 82.81% (p < 0.001), respectively as compared to control (Figure 4B). Exposure of osteoblasts to 1 nM of E2 increased the mineralization content to 68.85% (p < 0.001) as compared to control. Increased mineralization is synonymous to increased calcium deposition. The ability to form an extracellular matrix that can undergo regulated mineralization is the ultimate phenotypic expression of an osteogenic tissue [33]. PG treatment increased the calcium content notably in the mineralized matrix at 21 days of osteoblasts when compared with untreated cells. In this context, the phytoestrogen genistein has been previously found to stimulate bone mineralization *in vitro* [34].

### Effect of PG on cellular DNA content and cell cycle distribution of osteoblasts

**Figure 5:**
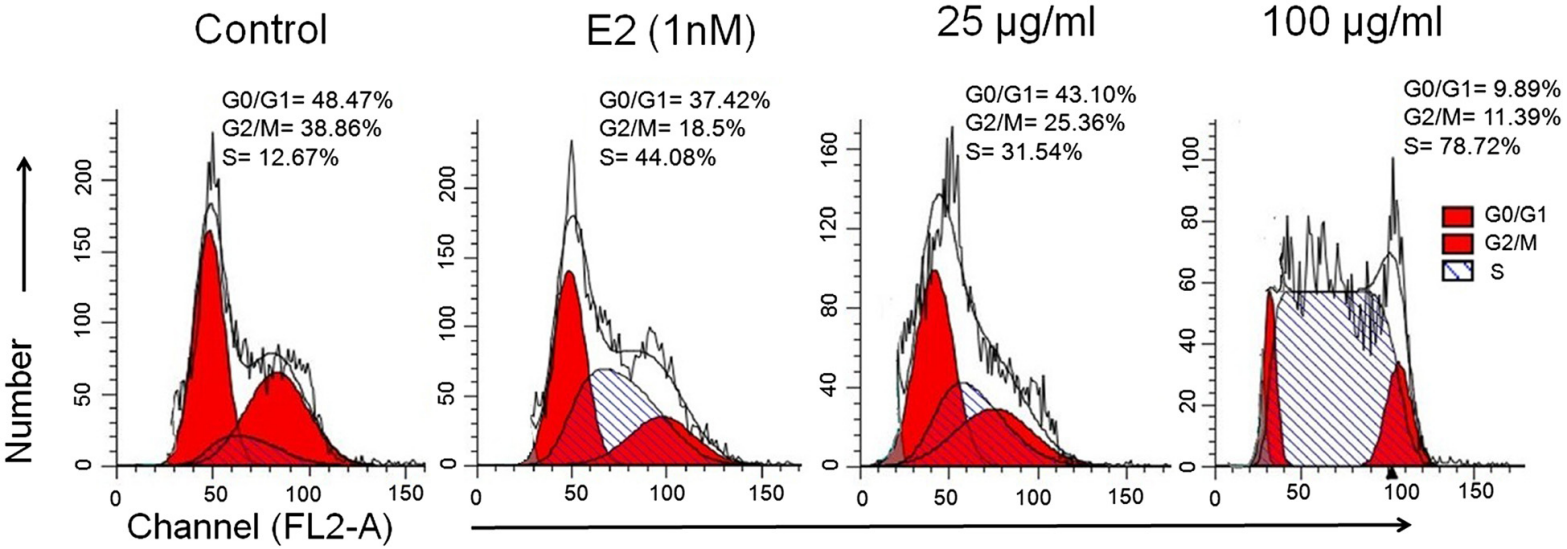
**Analysis of DNA content by flow cytometry.** Pictorial graph showing the proportion of osteoblastic cells in different phases of cell cycle stained with PI dye of treated with 1 nM of E2 as a positive control and 25 and 100 μg/ml of PG extract at 48 h.

The cellular DNA content and proportion of cells in different phases of the cell cycle was analyzed by using flow cytometry. Osteoblastic cells were treated with one lower (25 μg/ml) and one higher concentration (100 μg/ml) of the PG extract for 48 h and observed the different phases of the cell cycle. As evident from the results (Figure 5), a normal distribution of cell cycle was observed in the control group. At 25 μg/ml of PG extract, the accumulation of cells (DNA content) in S phase was sharply increased from 12.67 to 31.54% as compared to control. Moreover, 100 μg/ml concentration of the extract resulted to an incredible increment of DNA content in S phase by 78.72% as compared to control. Exposure of osteoblasts to 1 nM of E2 increased the DNA content in S phase by 44.08% greater at 25 μg/ml of PG treatment. These data indicate that the PG extract induce the cell proliferation by accumulating the DNA content in S phase of the cell cycle. A similar study has reported that estradiol induced the expression of estrogen receptor by promoting S phase of cell cycle in human osteosarcoma cell lines and pilose antler polypeptides promoted chondrocytes proliferation by accelerating cell cycle progression in S phase [35,24].

### Effect of PG on osteogenic gene runx2 expression

**Figure 6:**
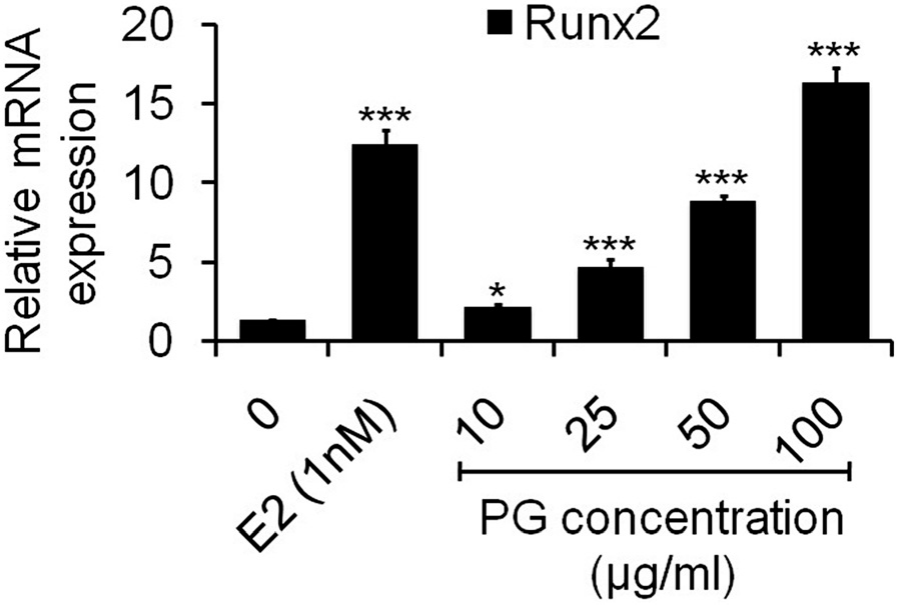
**Analysis of mRNA levels of Runx2 gene by qPCR.** Osteoblasts were treated with (10–100 μg/ml) of PG for 48 h. qPCR for Runx2 mRNAs was performed. At each concentration, PG increased the mRNA levels when compared to control. Fold changes in mRNA levels are indicated by the numbers derived after normalizing with GAPDH mRNA levels used as an internal control. Values are obtained from three independent experiments in triplicate and expressed as mean ± SEM; ^*^p < 0.05, ^*^p < 0.01 and ^***^p < 0.001 as compared with control.

To determine these results could be extended to another protein present in the organic bone matrix and effect of PG on the expression of Runx2 gene. Runx2 is a noncollagenous, highly conserved transcription factor involved in the regulation of mineralized matrix of bone. Increased expression of osteogenic gene was observed by qPCR in osteoblasts treated with PG extract at 48 h. As cleared from results (Figure 6), 10 μg/ml of PG extract significantly elevated the Runx2 expression as compared to control (p < 0.05). Moreover, concentrations 25, 50 and 100 μg/ml of PG increased the remarkable Runx2 level in a dose dependent manner (p < 0.001) as compared to control. It has been reported that Runx2 bonds to the osteoblast specific cis acting element which is found in the promoter region of all major osteoblast specific genes like osteocalcin, type-I collagen, BSP, OPN, ALP and control their expression [36]. Hence, it is reasonable to speculate that the estrogenic compounds such as β-sitosterol present in PG could have acted in a manner to induce mRNA expression of ALP, collagen and their protein levels and consequently increased Runx2 gene expression in osteoblastic cells. Furthermore, flavonoids have also been shown to increase the expression of Runx2 [37,38]. Thus, β-sitosterol and flavonoids present in PG might be responsible for the increased expression and transcriptional activity of Runx2.

## Conclusion

The present study showed that osteogenic potential of PG extract in primary calvaria osteoblasts is based on two salient features. (1) Cytochemical studies including cell proliferation, ALP stain, collagenation, matrix mineralization and DNA content in the S phase of the cell cycle in osteoblasts are the key parameters, which favor the osteogenic potential of PG. (2) A molecular marker Runx2 is a highly conserved osteogenic transcription factor involved in the regulation of bone cells proliferation and differentiation, which is also favors the osteogenic nature of PG.

Even though E2 used as a positive control shows the slightly higher effect on the proliferation, differentiation, collagenation, mineralization, DNA content and Runx2 gene expression of osteoblasts than lower doses, however, the side effects of the long-term use of estrogen, such as a higher incidence of endometrial cancer, cardiovascular disease and breast carcinoma, could not be ignored.

Thus, the results from this study suggest that PG promotes the function of osteoblasts and plays an important role in remodeling of the bone, which indicated that it may be one of anti-osteoporotic herbal candidate free from side effects. Accordingly, PG might be useful for alternative pharmacological agent of osteoporosis and skeletal tissues may benefit from the consumption of PG.

## Abbreviations

ALP: Alkaline phosphatase
α-MEM: Alpha modified minimum essential medium
E2: 17β-estradiol
BMPs: Bone morphogenetic proteins
Cbfα1: Core-binding factor alpha-1
DMSO: Dimethyl sulfoxide
ELISA: Enzyme linked immunosorbent assay
FBS: Fetal bovine serum
FDA: Food and drug administration
HRT: Hormone replacement therapy
MTT: (3-(4,5-dimethylthiazol-2-yl)-2,5-diphenyltetrazolium bromide)
PBS: Phosphate buffered saline
PG: *Punica granatum*
pNPP: *p*-nitrophenyl phosphate
PTH: Parathyroid hormone
PI: Propidium iodide
qPCR: Quantitative real-time PCR
Runx2: Runt-related transcription factor 2
SEM: Standard error mean
SERMs: Selective estrogen receptor modulators
TGFβ: Transforming growth factors
